# A Chemometry of *Aldrovanda vesiculosa* L. (Waterwheel, Droseraceae) Populations

**DOI:** 10.3390/molecules26010072

**Published:** 2020-12-25

**Authors:** Bartosz J. Płachno, Maciej Strzemski, Sławomir Dresler, Lubomír Adamec, Kamila Wojas-Krawczyk, Ireneusz Sowa, Anna Danielewicz, Vitor F. O. Miranda

**Affiliations:** 1 Department of Plant Cytology and Embryology, Institute of Botany, Faculty of Biology, Jagiellonian University in Kraków, Gronostajowa 9 St. 30-387 Cracow, Poland; 2 Department of Analytical Chemistry, Medical University of Lublin, Chodźki 4a, 20-093 Lublin, Poland; i.sowa@umlub.pl; 3 Department of Plant Physiology and Biophysics, Institute of Biological Science, Maria Curie-Skłodowska University, Akademicka 19, 20-033 Lublin, Poland; 4 Department of Experimental and Functional Morphology, Institute of Botany CAS, Dukelská 135, CZ-379 01 Třeboň, Czech Republic; lubomir.adamec@ibot.cas.cz; 5 Department of Pneumology, Oncology and Allergology, Medical University of Lublin, 20-090 Lublin, Poland; kamilawojas@wp.pl; 6 Department of Paediatric Orthopaedics, Medical University of Lublin, 20-093 Lublin, Poland; anna.danielewicz@umlub.pl; 7 Laboratory of Plant Systematics, Department of Applied Biology, Campus Jaboticabal, School of Agricultural and Veterinarian Sciences, UNESP-São Paulo State University, São Paulo CEP 14884-900, Brazil; vitor.miranda@unesp.br

**Keywords:** aquatic carnivorous plant, carnivorous plant, phylogeny, phenetics, plant taxonomy, critically endangered aquatic species

## Abstract

The genus *Aldrovanda* is a Palaeogene element containing a single extant species, *Aldrovanda vesiculosa* L. This aquatic carnivorous herb has a very wide range of distribution, natively covering four continents; however, it is a critically endangered aquatic plant species worldwide. Previous studies revealed that *A. vesiculosa* had an extremely low genetic variation. The main aim of the present paper is to explore, using chemometric tools, the diversity of 16 *A. vesiculosa* populations from various sites from four continents (Eurasia, Africa, Australia). Using chemometric data as markers for genetic diversity, we show the relationships of 16 *A. vesiculosa* populations from various sites, including four continents. Phytochemical markers allowed the identification of five well-supported (bootstrap > 90%) groups among the 16 populations sampled. The principal component analysis data support the idea that the strongly related African (Botswana) and Australian (Kimberley, NT, NW Australia) populations are the most distant ones, separated from the European and Asian ones. However, considering the five Australian populations sampled, three are nested within the Eurasian group. The chemometric data are correlated positively with the geographical distances between the samples, which suggests a tendency toward isolation for the most distant populations.

## 1. Introduction

**Figure 1:**
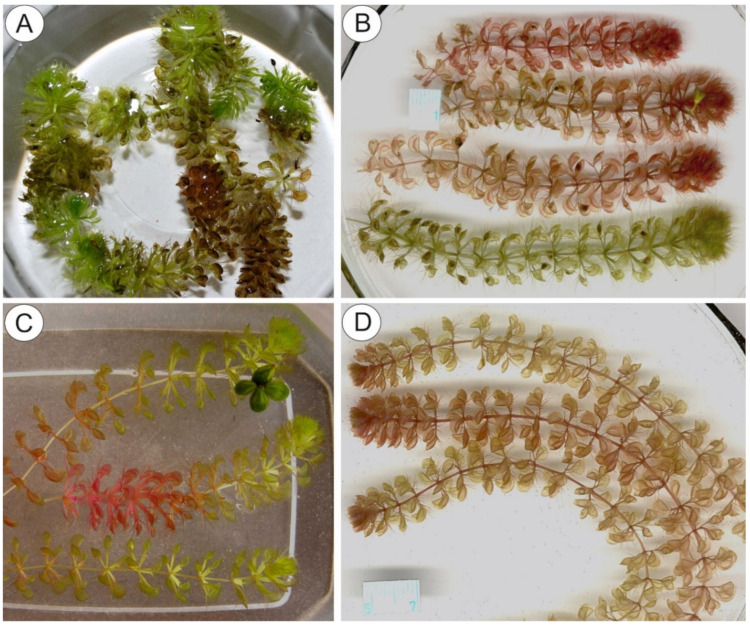
Diversity of *Aldrovanda vesiculosa* plants from different geographical origins. (**A**) Green plants from Lake Prespa, S Northern Macedonia. (**B**) Plants from Lake Baláta-tó, Somody county, SW Hungary. (**C**) Plants from Katherine, NT, N Australia. (**D**) Plants from Shallow Okavango delta, Botswana, Africa.

*Aldrovanda* L. is a monotypic genus containing a single extant aquatic species, *Aldrovanda vesiculosa* L. (Figure 1A–D), which produces leaves with snap-traps [1,2,3]. It belongs to the family Droseraceae and shares a common ancestor with the Venus’ flytrap (the monotypic *Dionaea*): both taxa are sister to the genus *Drosera* (c. 260 species). Both genera with snap-traps were derived from a common terrestrial ancestor that had flypaper-traps [4,5], and the lineages of *Aldrovanda* and *Dionaea* probably split about 48 million years ago [6]. It is accepted that *Aldrovanda* is a Tertiary (Palaeogene) element and that the recent *A. vesiculosa* is a relict species [3,4,7,8]. Despite its very wide range, it is an extremely rare aquatic plant species, possible due to several bottleneck events. This species is under severe threat, classified as ‘Endangered’ by The IUCN Red List of Threatened Species [9], and is gradually disappearing due to human impact and a lack of suitable habitats (eutrophication of sites from adjacent agriculture, fishery or the municipal pollution or drying of habitats [3,8,10]). Additionally, the “*Aldrovanda* disease”, probably caused by several *Phytopythium* and *Pythium* (Oomycetes) species, is harmful for the plants and may kill ex vitro and ex situ cultivated plants [11]; it can thus hinder the effort to conserve this endangered species.

Darwin [12] called *Aldrovanda* ‘*a miniature, aquatic Dionaea*’ and was probably the first researcher who discovered the carnivorous nature of this plant. *Aldrovanda vesiculosa* actively captures small aquatic animals: Crustacea, Ostracoda, Arachnida, fine larvae of Nematocera and Ephemeroptera, and fine Mollusca [13,14]. Anatomical and biophysical aspects of the *A. vesiculosa* trap function have recently been studied in detail [15,16,17,18,19,20]. A recent genomic study showed that, even with the evolution of the complex apparatus for carnivory, *Aldrovanda* and its sister genera (*Dionaea* and *Drosera*) are among the gene-poorest vascular plants so far found, with ~25,000 genes [5].

The aquatic habitat and worldwide distribution of *A. vesiculosa* across several climatic zones (four continents; introduced to North America), together with variations in characters such as anthocyanin production (Figure 1A–D, Table 1), growth pattern, turion formation, and the timing and shape of capsules, might suggest that *A. vesiculosa* has a high level of genetic variation [3,8]. However, all studies show an apparent “genetic paradox” as *A. vesiculosa* has a very low genetic diversity, so that even very distant and disjunct populations are genetically reminiscent of a monoclone [21,22,23,24].

Although the chemometric analysis supported by pattern recognition techniques has been accepted as a routine approach in the quality control and discrimination of herbs and herbal medicines [25], a comparison of phytochemical fingerprint profiles can also be a useful tool for clustering and improving the systematic classification [26]. Studies of the last decade show that chemometric techniques used for the exploration of multivariate data, obtained from an analysis of complex samples including plant matrices, are very useful in the verification and classification of plant families, species or populations [27,28,29,30]. Moreover, it was shown that phytochemical markers could be valuable in plant classification, including Droseraceae [31,32]. More recent investigations have also shown the usefulness of phytochemical markers in the chemotaxonomic classification of the *Drosera* species [33]. The authors proved that flavonoids and ellagic acid derivatives in *Drosera* were generally congruent with phylogeny based on cpDNA *rbc*L sequences and a morphology-based classification within the genus.

To the best of our knowledge, this is the first report describing the classification of *A. vesiculosa* accessions from different natural populations based on the chemometric analysis of gas chromatogram profiles. Using the chemometric data as markers for genetic diversity, we aim to reveal the relationships of 16 *A. vesiculosa* populations from various sites from four continents (Eurasia, Africa, Australia), comparing them with the literature data based on DNA [22,23,24], and we present some discussions about the phylogeography of world populations.

## 2. Results

**Figure 2:**
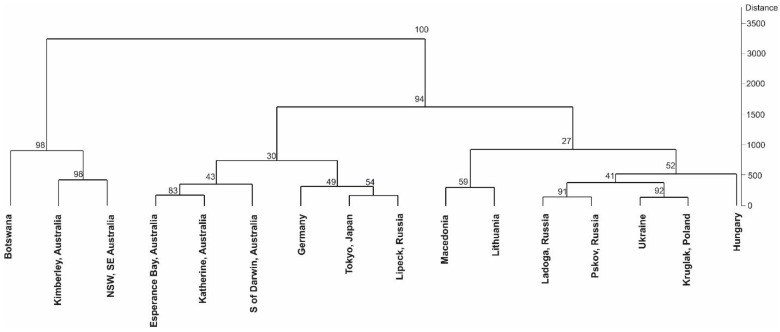
Hierarchical clustering dendrograms with bootstrap values based on the Euclidean distances with the Ward’s method are shown.

**Figure 3:**
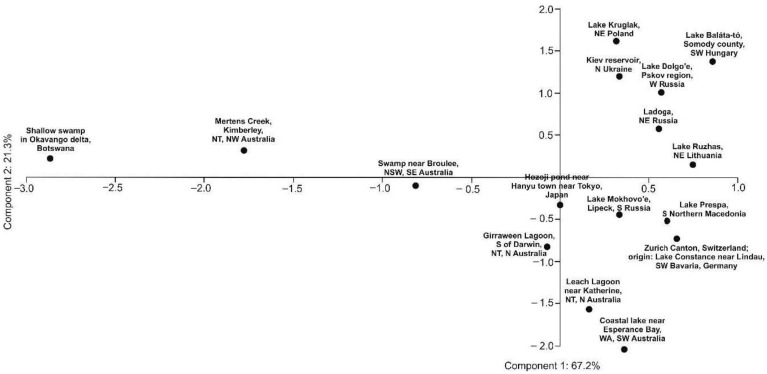
Principal component analysis of the GC phytochemical profiles of *Aldrovanda vesiculosa* populations. The numbers at the component names show the percentage of the explained variance.

The obtained GC-FID results and performed chemometric analysis allowed the identification of five well-supported (bootstrap > 90%) groups (Figure 2, Supplementary Figure S1) among the 16 populations sampled. The principal component analysis (PCA) supported that the African (Botswana) and the Australian (Kimberley, NT, NW Australia) are the most distant populations, separated from the European and Asian populations (Figure 3).

The Mantel test resulted in a significant positive correlation between the two distance pairwise matrices: the matrix of geographic distances and that of chemometric data (r = 0.3350; *p* = 0.008).

## 3. Discussion

The genus *Aldrovanda* has a complicated history, and several fossil species are known [7]. In the case of *A. vesiculosa*, there are two main theories of how the modern distribution range of this species has arisen. According to Huber [34] and Sculthorpe [35], *A. vesiculosa* survived glaciations in Africa and Australia and later recolonized Europe. Thus, African and Australian plants should be much older than European ones. However, Cross [3] suggests that *Aldrovanda* survived glaciations at refugia in Southern Europe and only colonized Africa and Australia later, about 50,000–200,000 years ago. According to Cross, water bird migration probably dispersed *Aldrovanda* from Europe through Asia and finally to Australia. However, Huang et al. [36] has recently described seeds of *A. vesiculosa* from the late Miocene in Southwest China, and this could indicate that in Asia, *A. vesiculosa* occurred much earlier than previously thought. But these seeds may represent another related fossil species, and this problem requires further research. One of the most important recent arguments for the origin of modern *A. vesiculosa* in temperate Europe and/or Asia is based on the formation of turions: all extant temperate populations and two tropical Australian populations (Katherine, N.T.; Armidale, N.S.W.) form dormant turions, while the remaining Australian and African populations do not [8]. Moreover, in hybrids, turion formation is of a dominant nature. These facts may suggest that the turion-forming ability, usual in temperate populations, could be plesiomorphic, while the (sub)tropical African and Australian populations could be derived and that the turion formation ability has been silenced by selective gene expression [37] or even lost by mutations. A similar question can be raised about the evolution of the red anthocyanin pigmentation. Further studies are needed to test the Eurasian origin hypothesis.

Our chemometric analysis showed that the African population from Botswana and two Australian populations (Kimberley, NT, NW Australia and NSW, SE Australia) formed a group (bootstrap = 98%) that was sister to another 13 populations (Figure 2). Furthermore, the principal component analysis (PCA) supported the idea that the African (Botswana) and the Australian (Kimberley, NT, NW Australia) populations were the most distant from the others (Figure 3).

Our results do not support the clear splitting between the red (anthocyanin-producing without turions), represented by African and Australian populations, and green (with turions) lineages, represented by Asian and European populations. The presence of Australian populations (Esperance Bay, Katherine and South of Darwin) clustered within European and Asian populations suggests that different lineages reached Australia, possibly resulting from more than one colonization event. To consider Africa as a possible cradle of *Aldrovanda vesiculosa* [22] seems unreasonable without a rooted tree or more sampled populations and a phylogenetic/phylogeographic perspective. Therefore, for the same reasons, it is also impracticable to infer the colonization direction based on our results, whether it was from Eurasia to Africa or vice versa. A rooted tree could solve this dilemma, with the inclusion of more *Aldrovanda* populations (particularly from Africa and Asia) with an outgroup.

The Mantel test revealed a significant positive correlation between geographic distances and chemometric data (r = 0.3350; *p* = 0.008), indicating that geographically closer populations tended to be more related but that with increasing geographical distances, the populations were more isolated. Considering the fact that the accessions originated from natural populations, which were cultivated in the same location (Třeboň, Czech Republic) and under the same conditions for years, it is reasonable to suppose that the metabolites measured in this study are genetically determined. However, the signal provided by the phytochemical markers was not high and allowed the identification of only five well-supported (bootstrap > 90%) groups (Figure 2).

According to previous population genetic studies on *Aldrovanda vesiculosa*, the populations presented a very low genetic diversity [21,22,23,24]. This supports the hypothesis of the recent origin of the species and a severe bottleneck [24] with the recent gene flow via transportation by migratory birds [38]. Additionally, the low sexual reproductive capability, usually with flowers setting very few fertile fruit [39,40], and the predominant clonal reproduction of *A. vesiculosa* must have contributed to the low population diversity.

Hoshi et al. [23] obtained the same haplotype for the ITS region (rDNA) with populations from different continents. This result seems surprising, given that the same genome can host thousands of ITS copies and that the concerted evolution commonly fails to homogenize the different paralogues [41,42,43]. However, the PCR protocol can be selective and amplify specific haplotypes restricted by the stringency of reactions [44], which could explain the single ITS obtained. Thus, the conservative result for ITS was possibly a matter of sampling, not only via the few populations sampled but also via the selective DNA amplification.

To better understand and solve the “*Aldrovanda* paradox”, more effort will be needed. Different hypotheses have been proposed so far (e.g., recent origin of the species, recent gene flow, severe bottleneck, low mutation rate), but more robust data are necessary to test them. For that, a NGS (Next-generation sequencing) approach, based on RADseq or GBS (Genotyping-by-sequencing), for example, could explore the genomes more deeply, particularly with the sampling of more populations from different continents (particularly from Africa and Asia). Unveiling genomic traits in more detail could bring answers about the genetic diversity, mutation rates [24] and colonization events that drove the present distribution and help to shine light on the natural history of *Aldrovanda vesiculosa*.

## 4. Materials and Methods

### 4.1. Plant Material and Growth Conditions

The material from 16 populations of *Aldrovanda vesiculosa* L. (Table 1) was taken from the collection at the Institute of Botany CAS at Třeboň, Czech Republic. The plants were grown in 3-l aquaria, which stood outdoors in a 2.5-m^2^ plastic container filled with water for cooling or indoors on a window ledge, and sedge litter was used as a substrate to create a dystrophic environment [22,24]. The plants from each population were grown separately and were propagated strictly vegetatively.

### 4.2. Sample Preparation

Fresh clean material was blotted dry, frozen at –25 °C and lyophilized. The powder of the plants (50 ± 1 mg DW) was extracted in methanol (10 mL) in an ultrasonic bath at room temperature for 30 min. The extracts were centrifuged at 10,000 g for 10 min and filtered through a 0.22-µm nylon membrane filter.

### 4.3. Gas Chromatography Analysis

Gas chromatography with a flame ionization detection (GC-FID) analysis was conducted according to a previously published methodology [45] with minor modifications. The extracts were analyzed using an Agilent 7820A GC-FID system with a HP-5 capillary column (30 m × 0.32 mm; 0.25 µm film thickness), a split-splitless injector and OpenLAB CDS software (Agilent Technologies, Santa Clara, CA, USA). The oven temperature was programmed from 70 °C to 230 °C at a rate of 20 °C/minute and then held at 290 °C for 10 min. The temperature of the injector and interface was 250 and 300 °C, respectively. The injection volume was 2.5 µL (split ratio 1:50). Helium was used as a carrier gas at a flow rate of 2 mL/min.

### 4.4. Preprocessing of Data and Chemometric Analysis

The raw data obtained from the gas chromatography before the multivariate analysis were subjected to preprocessing according to the method described earlier [28,29]. Chemometric analysis GC data were imported into SpecAlign (SpecAlign ver. 2.4.1., University of Oxford), and bin processing was performed. The baseline and the noninformative part of the chromatograms from 0 to 1.9 min contained a large peak from methanol and were removed. Afterwards, the reduced data were aligned using a Fast Fourier Transform method with 20 maximum shift parameters [46]. After the pretreatment of GC data, 16 × 2089 (samples × retention times) datasets were achieved. The obtained data matrix (CDM) was used to construct dendrograms based on the Euclidean distance and Ward’s method with 1000-bootstrap resampling. Moreover, a principal component analysis (PCA) was carried out. Both the PCA and dendrograms were constructed using PAST4.02 software [47].

### 4.5. Mantel Test

We tested for isolation by distance using the Mantel test [48]. For that, we used the Python implementation MantelTest v.1.2.10 [https://github.com/jwcarr/MantelTest]. The Mantel test analyzes the significance of correlation between two data matrices; for this study, we used the geographical pairwise distance matrix (GDM), calculated with the geographical coordinates for each population (Table 1) using the program Geographic Distance Matrix Generator (version 1.2.3) [49], and the pairwise distance matrix resulting from the CDM matrix with the chemometric data. The Mantel test was calculated with 10,000 permutations.

## 5. Conclusions

Our results, based on chemometric data, provide a signal to partially distinguish African and Australian from Asian and European populations; however, considering the five Australian populations sampled, three are nested within the Eurasian group.

The chemometric data were positively correlated with the geographical distances between the samples, which suggests a tendency of isolation for distant populations.

## Figures and Tables

**Table 1:** List of 16 accessions of *Aldrovanda vesiculosa* used for phytochemical analyses.

Origin, Site, Country	Geographical Coordinates	Color	Turion Formation	Turion Formation Time
Lake Kruglak, NE Poland	53°54′ N 23°19′ E	green	Yes	Sep
Lake Ruzhas, NE Lithuania	55°30′ N 25°28′ E	green	Yes	Early Sep
Ladoga, NE Russia	60°29′ N 32°57′ E	green	Yes	Early Aug
Lake Dolgo’e, Pskov region, W Russia	56°08′ N 28°22′ E	green	Yes	Late Aug
Lake Mokhovo’e, Lipeck, S Russia	52°24′ N 39°34′ E	green	Yes	Sep
Kiev reservoir, N Ukraine	51°03′ N 30°25′ E	green	Yes	Sep
Lake Baláta-tó, Somody county, SW Hungary	46°19′ N 17°12′ E	red	Yes	Sep
Zurich Canton, Switzerland; origin: Lake Constance near Lindau, SW Bavaria, Germany	47°34′ N 9°41′ E	green	Yes	Sep
Lake Prespa, S Northern Macedonia	41°01′ N 20°59′ E	green	Yes	Sep
Hozoji pond near Hanyu town near Tokyo, Japan	36°12′ N 139°42′ E	green	Yes	Sep
Shallow swamp in Okavango delta, Botswana	19°33′ S 23°13′ E	red	No	-
Swamp near Broulee, NSW, SE Australia	35°35′ S 150°09′ E	red	No	-
Girraween Lagoon, S of Darwin, NT, N Australia	12°31′ S 131°05′ E	red	No	-
Leach Lagoon near Katherine, NT, N Australia	14°38′ S 132°37′ E	red	Yes or No	Oct–Nov
Mertens Creek, Kimberley, NT, NW Australia	14°50′ S 125°41′ E	red	No	-
Coastal lake near Esperance Bay, WA, SW Australia	33°48′ S 121°49′ E	red	No	-

## Data Availability

The data presented in this study are available on request from the corresponding author.

## Supplementary Materials

The following are available online: Figure S1. GC-FID chromatographic profiles of Aldrovanda vesiculosa populations. On the right, hierarchical clustering dendrograms with bootstrap values based on the Euclidean distances with the Ward’s method are shown.
