# Supplementary material for: A Chemometry of Aldrovanda vesiculosa L. (Waterwheel, Droseraceae) Populations
Source: Molecules. 2020 Dec 25;26(1):72. doi: 10.3390/molecules26010072 (PMC7795913; doi:10.3390/molecules26010072)
Supplement: Supplementary file 1 [file molecules-26-00072-s001.pdf]

molecules

SUPPLEMENTRAY MATERIALS

A Chemometry of *Aldrovanda Vesiculosa* L. (Waterwheel, Droseraceae) Populations

Bartosz J. Płachno [1,*], Maciej Strzemski [2,*], Sławomir Dresler [3*], Lubomír Adamec [4], Kamila Wojas-Krawczyk [5], Ireneusz Sowa [2], Anna Danielewicz [6] and Vitor F. O. Miranda [7]

[1] Department of Plant Cytology and Embryology, Institute of Botany, Faculty of Biology, Jagiellonian University in Kraków, Gronostajowa 9 St. 30-387 Cracow, Poland; bartosz.plachno@uj.edu.pl (B.J.P.); https://orcid.org/0000-0001-5579-5101
[2] Department of Analytical Chemistry, Medical University of Lublin, Chodźki 4a, 20-093 Lublin, Poland: maciej.strzemski@poczta.onet.pl (M.S.), i.sowa@umlub.pl (I.S.)
[3] Department of Plant Physiology and Biophysics, Institute of Biological Science, Maria Curie-Skłodowska University, Akademicka 19, 20-033 Lublin, Poland; slawomir.dresler@poczta.umcs.lublin.pl (S.D.);
[4] Department of Experimental and Functional Morphology, Institute of Botany CAS, Dukelská 135, CZ-379 01 Třeboň, Czech Republic; lubomir.adamec@ibot.cas.cz (L.A.);
[5] Department of Pneumology, Oncology and Allergology, Medical University of Lublin, 20-090 Lublin, Poland kamilawojas@wp.pl (K.W-K.);
[6] Department of Paediatric Orthopaedics, Medical University of Lublin, 20-093 Lublin, Poland (A.D.); anna.danielewicz@umlub.pl;
[7] UNESP - São Paulo State University, School of Agricultural and Veterinarian Sciences, Department of Applied Biology, Laboratory of Plant Systematics, Campus Jaboticabal, CEP 14884-900, SP, Brazil; vitor.miranda@unesp.br (V.M.);
* Correspondence: maciej.strzemski@poczta.onet.pl; bartosz.plachno@uj.edu.pl; slawomir.dresler@poczta.umcs.lublin.pl.

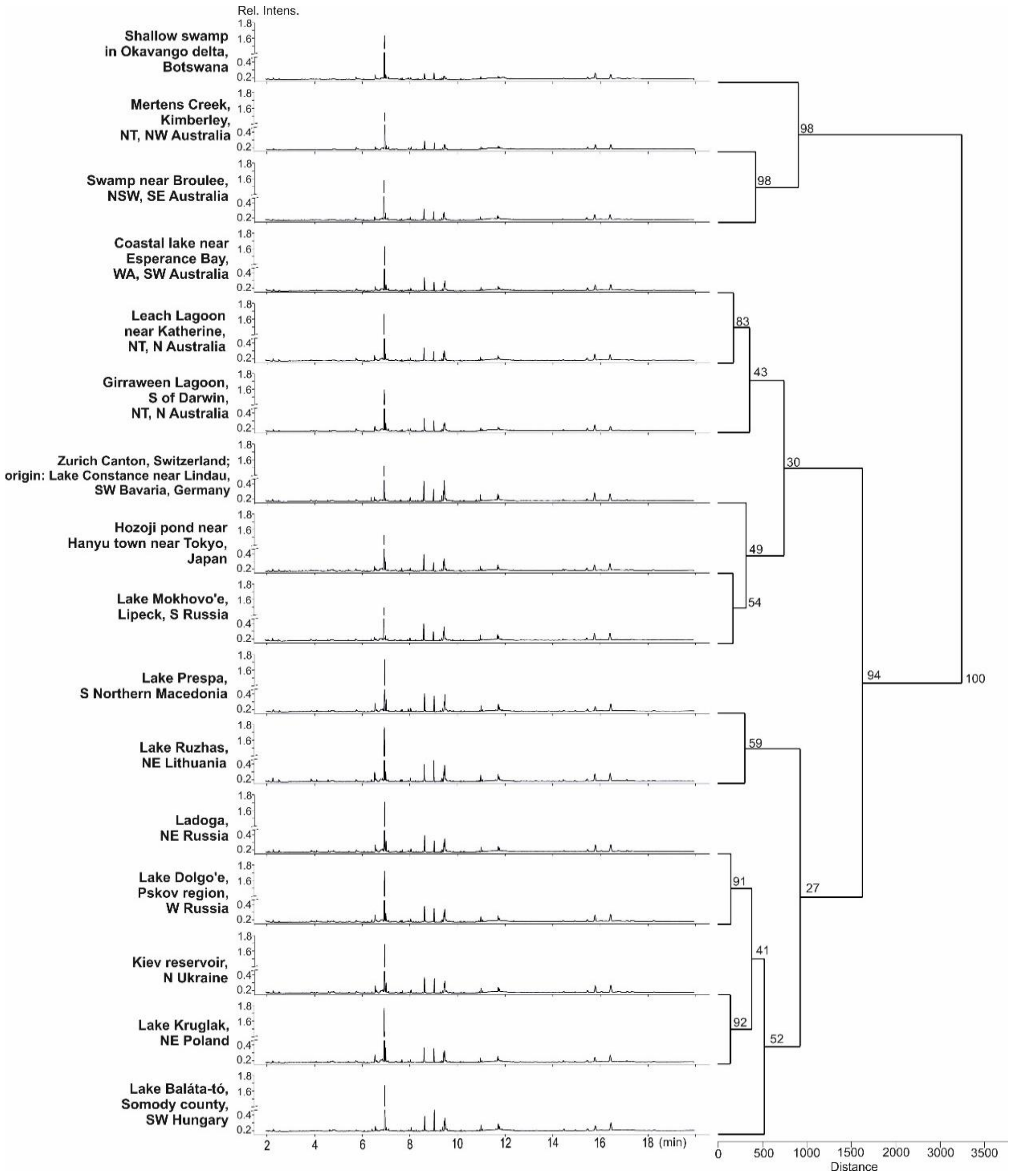

Figure S1. GC-FID chromatographic profiles of *Aldrovanda vesiculosa* populations. On the right, hierarchical clustering dendrograms with bootstrap values based on the Euclidean distances with the Ward's method are shown.
